# Biomechanical and Kinematic Gait Analysis in Lower Limb Amputees: Cross-Sectional Study

**DOI:** 10.2196/67022

**Published:** 2025-07-29

**Authors:** Natali Olaya Mira, Luz Marina Gómez Hernández, Carolina Viloria Barragán, Manuela Monsalve Montes, Isabel Cristina Soto Cardona

**Affiliations:** 1 Grupo de Investigación e Innovación Biomédica GI2B, Facultad de Ciencias Exactas y Aplicadas, Instituto Tecnológico Metropolitano, Calle 73 No. 76A - 354, Vía al Volador (Bloque I, Segundo piso, oficina 7), Medellin, 050034, Colombia, 57 6044405100 ext 5166; 2 Escuela de Ingeniería, Arquitectura y Diseño, Universidad Tecnológica de Bolívar, Parque Industrial y Tecnológico Carlos Vélez Pombo Km 1 Vía Turbaco,Cartagena, Colombia, +57 605 6931919

**Keywords:** amputee, gait analysis, body weight distribution, gait symmetry, prosthetics

## Abstract

**Background:**

The quantification of gait parameters in amputees facilitates the assessment of their performance with prosthetic devices. These parameters often depend on measurements based on anatomical aspects that vary across different types of lower limb amputations.

**Objective:**

This study aimed to investigate body weight distribution, and gait symmetry, quality, and propulsion, as well as pelvic kinematics in the amputee population.

**Methods:**

The EcoWalk baropodometry platform was used to measure plantar pressure, and the G-Walk inertial sensor was used for accelerometry measurements in 29 unilateral lower limb amputees.

**Results:**

Values were estimated for each variable under analysis, and the findings were categorized by the level of amputation. All variables exhibited normal distribution within each group under analysis , except for the symmetry index in above-knee (AK) amputees (*P*=.03). Regarding the body weight distribution (*P*=.11), velocity (*P*≥.99), propulsion (*P*=.38), and quality index (*P*=.10) of the amputated limb; no significant differences were observed between the AK and below-knee (BK) amputees. The most significant deviation was noted in pelvic obliquity, which was greater in AK amputees compared to BK amputees.

**Conclusions:**

The values reported for the variables under analysis may enable the establishment of more precise reference levels for the amputee population, thereby contributing to a more accurate diagnostic process and aiding prosthetic fitting.

## Introduction

Lower limb amputation causes drastic changes in basic locomotion patterns [1]. Currently, these patterns are analyzed using a wide variety of methods [2], with a focus on different variables that describe their impact on gait conditions and normal posture. Evaluating these conditions is of paramount importance because restoring a normal gait constitutes a key objective in the physical rehabilitation of amputees. Moreover, such assessments may suggest various modifications to prosthetic devices [3].

Notably, spatiotemporal, kinematic, and kinetic variables dominate several gait studies [3-5]. Among these, the two-minute walking test (2MWT) is one of the most commonly used tests for assessing amputees’ gait performance [6,7]. Nevertheless, given the particular gait conditions of amputees, pivotal emphasis is placed on aspects such as body weight distribution (BWD) and the propulsion, symmetry, and quality indices when analyzing gait parameters.

BWD denotes how the body weight is supported by the prosthetic device and sound limb in the orthostatic position [1]. The indices, for their part, are related to gait parameters. Specifically, the propulsion index describes individuals’ ability to push the center of mass forward during the single support stance phase [8]. The symmetry index represents the difference between the value (expressed as a percentage) of the sound limb and that of the amputated limb in the stance or swing phases [9]. Finally, the quality index, unlike the symmetry index, expresses a characteristic of a single extremity. It evaluates individuals’ ability to correctly divide their own gait cycle between the sound and amputated limb steps [10].

Although reference values for these variables exist for healthy individuals, there are limited data reported for the amputee population, except for the 2MWT. The reference values for such tests have been provided considering different amputation-related demographic characteristics, with, for instance, a reported distance of around 140 m [11].

In healthy individuals, body weight is evenly distributed between both lower limbs [12]. Nonetheless, this condition, changes in lower limb amputees. In the case of unilateral transfemoral amputees, a significant proportion of their weight is transferred to the intact limb during most movements [13]. In the case of transtibial amputees, Fontes et al [1] reported that, during the early stages of rehabilitation, the nonamputated limb supports up to 60% of the body weight.

Furthermore, walking patterns in healthy individuals are symmetrical, deviating only slightly from the ideal values. However, in a pathological gait, an asymmetry between the lower limbs can be noticeably observed, which can have serious implications for the health of the intact limb [14]. In a study involving transfemoral amputees, Winiaski et al [9] used the symmetry index based on time measures and vertical force and reported a difference between the two lower limbs of less than 6% for normal gait. For transtibial amputees, the absolute asymmetry index has been found to be approximately 0.632 during knee flexion at loading response [15]. In general, gait among individuals with transfemoral amputation appears to be more asymmetric than among those with transtibial amputation [3].

Importantly, all these reference values reported in the literature for BWD and gait symmetry in amputees differ in the way these variables were estimated and the anatomical reference points. These variations make comparisons difficult, as amputees contend with muscle absence, coupled with the fact that they behave differently during standing and gait depending on the level of amputation.

In this study, accelerometry was used to calculate different indices based on generic gait parameters, which facilitate making comparisons between populations with gait impairments using reference values reported for healthy individuals. Additionally, these acceleration data can be used to evaluate pelvic kinematic patterns across different planes, providing insights into pelvic tilt, obliquity, and rotation during the gait cycle.

Given these considerations, this study aims to provide an estimate of BWD based on pressure calculations between the lower limbs during the standing position, pelvic kinematics, and gait measurements in above-knee (AK) and below-knee (BK) amputees. Notably, this analysis will be exclusively based on the gait cycle duration in each limb for the entire sample of amputees.

## Methods

### Recruitment

The study participants were 29 unilateral lower limb amputees aged 18 to 59 years. Inclusion criteria required participants to be independent walkers, capable of gait without any external technical aids. Patients who had undergone lower limb disarticulation and amputation surgery within the preceding 12 months were excluded from the study. The participants who met the inclusion criteria signed the corresponding informed consent.

Among the 29 participants, 14 had AK amputations and 15 had BK amputations, with an even distribution in terms of laterality and amputation level. The average age was 41.2 (SD 11.4) years, and the mean BMI was 25.3 (SD 4.5). On average, participants lived with amputated limbs for 11.8 (SD 10.4) years and had prior experience using prosthetic devices.

### Patient Instrumentation

**Figure 1.**
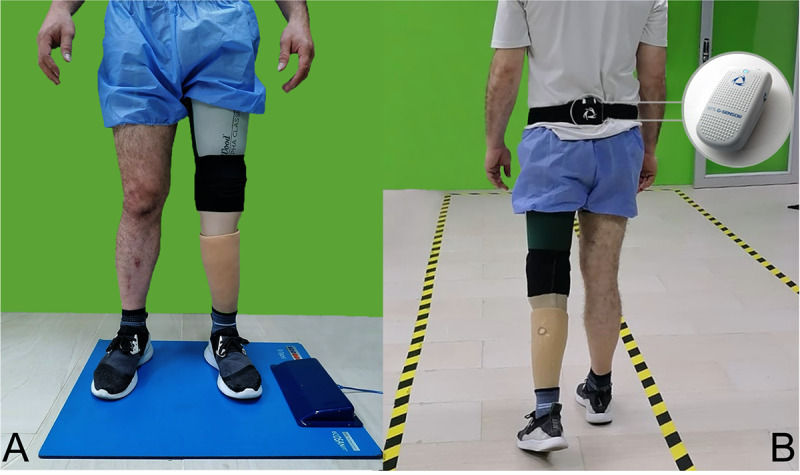
Experimental setup for gait assessment. (A) Plantar pressure recording during static standing; (B) Inertial sensor placement at the S1 level for dynamic gait analysis.

Participants were instructed to stand on a plantar pressure platform, starting at a fixed point with both feet placed shoulder-width apart, and to hold this position for 60 seconds (Figure 1A). Then, they were directed to walk back and forth on a walking course as quickly as possible, without stopping, for two minutes. For this activity, an inertial sensor was placed on the participant’s back at the level of the S1 vertebra (Figure 1B).

### Data Acquisition

The 2MWT was performed using a stopwatch and distance markers found in the facility. All equipment used for the assessments belonged to the Laboratory of Biomechanics and Rehabilitation of the Instituto Tecnológico Metropolitano (ITM), located in Medellín, Colombia.

BWD was measured using the EcoWalk portable plantar pressure platform (Ecosanit) with the EcoFoot software (version 4.0). This device comprises a 480 × 480 mm active matrix with 2304 sensors and an acquisition rate of 40 frames per second [16]. The pressure exerted by each foot activates the sensors, and the obtained data are used to calculate the distribution of body weight on the legs. BWD is determined as the difference between the weight distribution values of the sound and amputated limb. An ideal BWD would represent a difference tending towards 0% [12,17].

For the analysis of gait quality, the G-Walk inertial sensor (BTS Bioengineering) was used, along with the G-Studio software (version 3.2.25.0). This device operates wirelessly via Bluetooth 3.0 (Class 1.5, with a range of up to 60 m). In addition, it contains four sensors, each equipped with triaxial elements such as: an accelerometer (frequency range: 4‐1000 Hz), a magnetometer (maximum frequency: 100 Hz), and a gyroscope (frequency range: 4‐8000 Hz) [18]. The signal was acquired, and variables including velocity, cadence, and the overall symmetry index were calculated. Additionally, each limb’s stride length, propulsion, and quality index were estimated.

The symmetry index measures the difference between the sound and amputated limbs during stance or swing phases, with values between 75% and 100% indicating high symmetry. It is calculated by analyzing the anteroposterior (AP) acceleration signals from both sides, computing the mean normalized values, and determining the Pearson correlation coefficient. This correlation is then mapped to a 0 to 100 scale, where higher values indicate greater symmetry [18]. To calculate the symmetry index, the following formula is used:


$$Symmetry\;index=\frac{\left(\frac{\sum \left(AP_{s}-{\overset{-}{AP}}_{s}\right)\left(AP_{a}-{\overset{-}{AP}}_{a}\right)}{\sqrt{\sum {\left(AP_{s}-{\overset{-}{AP}}_{s}\right)}^{2}\sum {\left(AP_{a}-{\overset{-}{AP}}_{a}\right)}^{2}}}+1\right)x100}{2}$$

, where

AP is the anterior-posterior (AP) acceleration values for the sound (s) or amputated (a) limb throughout the gait cycle.

$\overset{-}{AP}$ is the mean AP acceleration value for the sound (s) or amputated (a) limb, respectively, representing the average acceleration over the gait cycle for each limb.

The propulsion index, is computed based on the gradient (in degrees) between the start and end of the monopodal support phase on the AP acceleration graph for each limb during gait [19]. The optimal value of this index is expected to be higher than 5.5°; this parameter describes the patient’s ability to fully accept body weight on a limb after the deceleration phase and push the center of mass forward on the opposite limb (acceleration phase). A higher propulsion index denotes greater progression capacity of the participant during the single-support phase.

**Figure 2.**
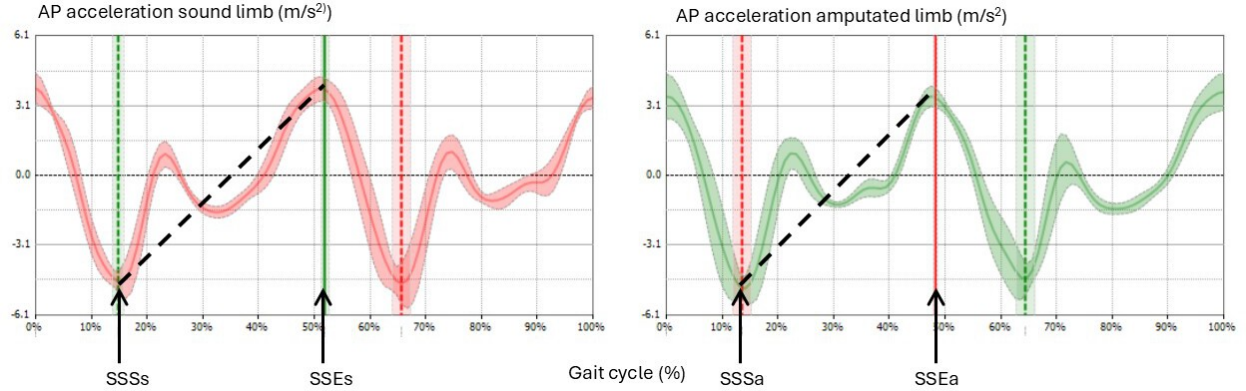
Acceleration graphs of the sound limb (right) and amputated limb (left) during gait. AP: anteroposterior; SSEa: single-support end for amputated limb; SSEs: single-support end for sound limb; SSSa: single-support start for amputated limb; SSSs: single-support start for sound limb.

First, the mean normalized AP acceleration signal is obtained separately for the left and right sides (Figure 2). Then, the single support start and single support end are identified for the sound (s) and amputated (a) limb. A line is traced between the acceleration values at these points, and its slope is computed for both limbs. The propulsion index is determined using the following formula:


$$Propulsion\;index={tan}^{-1}\frac{\Delta_{a}}{\Delta_{t}}$$

${∆}_{a}$ represents the difference in AP acceleration between the start and end of the single support phase for each limb and ${∆}_{t}$ represents the duration of this phase in seconds for each limb.

Finally, the quality index is calculated by adding the absolute percentage differences observed between the stance and swing phases for each limb. Ideally, a value of 100 is attained when the stance and swing phases of each limb correspond exactly to 60% and 40%, respectively, of the entire gait cycle [18]. It is calculated using the following expression:


$$Quality\;index=\;\left|\left|\left(\overset{-}{D_{stance}}-\;\overset{-}{D_{swing}}\right)-20\%\right|-100\%\right|$$

$\overset{-}{D_{stance}}$ corresponds to the mean stance phase duration as a percentage of the gait cycle, and $\overset{-}{D_{swing}}$ corresponds to the mean swing phase duration as a percentage of the gait cycle.

### Statistical Analysis

The Shapiro–Wilk test was used to assess normality of the data. For variables that met normality assumptions, the Student *t* test was applied, whereas the Wilcoxon test was used for those that did not. Data processing and figure generation were performed using R studio software (version 6.0; R Foundation for Statistical Computing), and the level of statistical significance for the entire study was set at α=.05.

### Ethical Considerations

This study was approved by the Bioethics Committee of the Universidad de Antioquia (approval record number 21-21-954; June 2, 2021) and conducted in accordance with the Declaration of Helsinki. All participants provided written informed consent prior to enrollment and were informed that they could withdraw from the study at any time without any consequences. All data collected were fully anonymized before analysis to ensure privacy and confidentiality. No identifiable images or personal data were collected or included in this publication or any supplementary materials. No compensation—monetary or otherwise—was provided to participants for their involvement in this study.

## Results

Knee braces were the most commonly used prosthetic suspension system among participants, followed by vacuum systems and belts.

**Figure 3.**
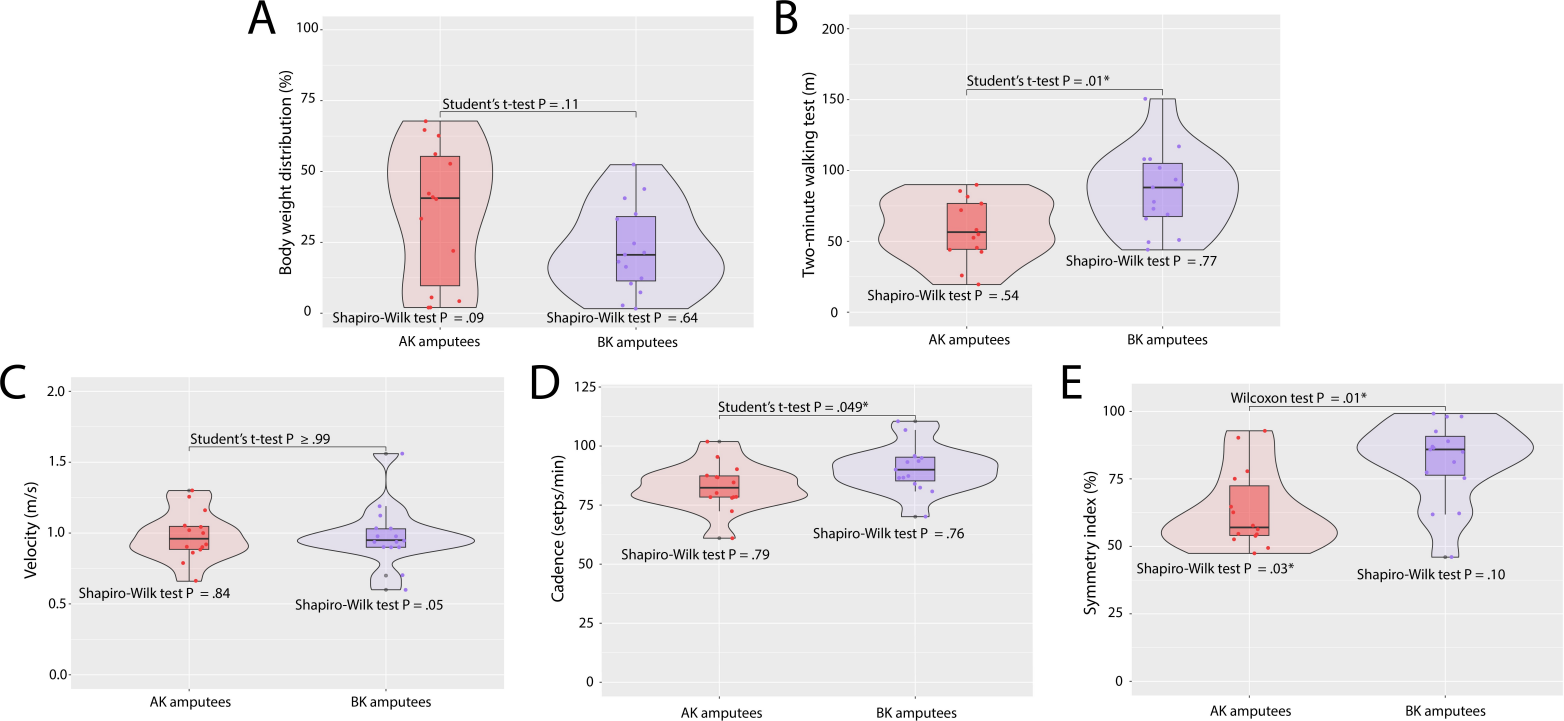
(A) BWD is the absolute value of the difference in weight distribution, ideally close to zero (0%); (B) 2MWT measures the distance covered in 2 minutes; a greater distance indicates better endurance and mobility; (C) Velocity refers to an individual’s walking speed, higher velocity reflects better functional performance; (D) Cadence measures steps per minute, with higher values indicating better mobility; (E) Symmetry index measures the difference between the sound and amputated limb, with 75% to 100% indicating high symmetry. *P<.05.

The estimated values for each variable under analysis were divided by the level of amputation (Table 1, Figures3  4). Table 1 shows the mean (SD) of the variables, while the figures display the quartiles, data dispersion, and confidence levels for normality and inferential tests. Also, mean (SD) pelvic kinematics across the entire gait cycle are shown in Figure 5. All variables exhibited normal distribution within each group under analysis, except for the symmetry index in AK amputees. No significant differences were observed between AK and BK amputees for BWD, velocity, or the propulsion and quality indices of the amputated limb. Although BK amputees exhibited a better BWD, with a value closer to zero, the asymmetry between both limbs still persisted in this group. Notably, weight loss as a result of amputation was not uniform among most amputees, which may have influenced these results.

**Table 1.** Distribution of variables between the two amputation groups.

Variables	Amputation groups	
	AK^a^, mean (SD)	BK^b^, mean (SD)
BWD^c^ (%)	35.5 (24.4)	22.7 (15.4)
2MWT^d^ (m)	59.0 (22.1)	85.8 (28.9)
Velocity (m/s)	0.98 (0.18)	0.98 (0.21)
Cadence (steps/min)	82.9 (10.0)	90.5 (10.2)
Symmetry index (%)	63.5 (14.7)	81.7 (15.1)
Propulsion (°)		
Amputated limb	6.1 (2.5)	6.9 (2.0)
Sound limb	3.4 (1.5)	5.8 (2.4)
Quality index (%)		
Amputated limb	81.0 (14.6)	88.8 (8.1)
Sound limb	80.4 (10.6)	91.6 (8.2)
Stride length (m)		
Amputated limb	1.60 (0.39)	1.32 (0.17)
Sound limb	1.60 (0.39)	1.32 (0.17)

a AK: above-knee.

b BK: below-knee.

c BWD: body weight distribution.

d 2MWT: two-minute walking test.

**Figure 4.**
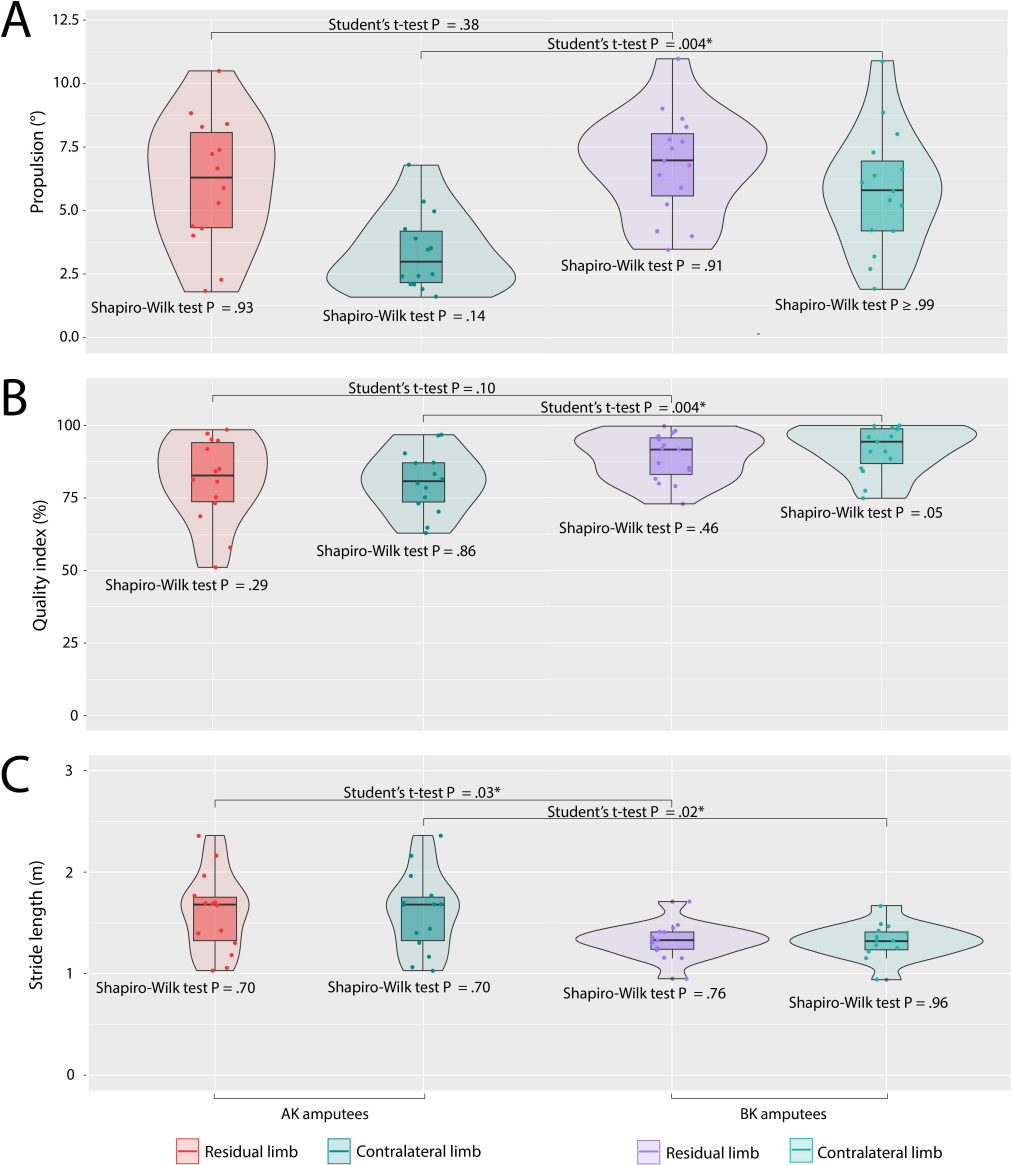
(A)Propulsion index describes the ability to bear weight on one limb after deceleration and propel the body forward during acceleration, with higher values reflecting better progression; (B) Quality index measures how balanced the stance and swing phases are during a step, with a perfect score of 100 indicating an ideal distribution; (C) Stride length measures distance covered between two consecutive initial contacts of the same foot during walking. Although influenced by anthropometry, stride length is typically between 1 and 2 meters. *P<.05.

**Figure 5.**
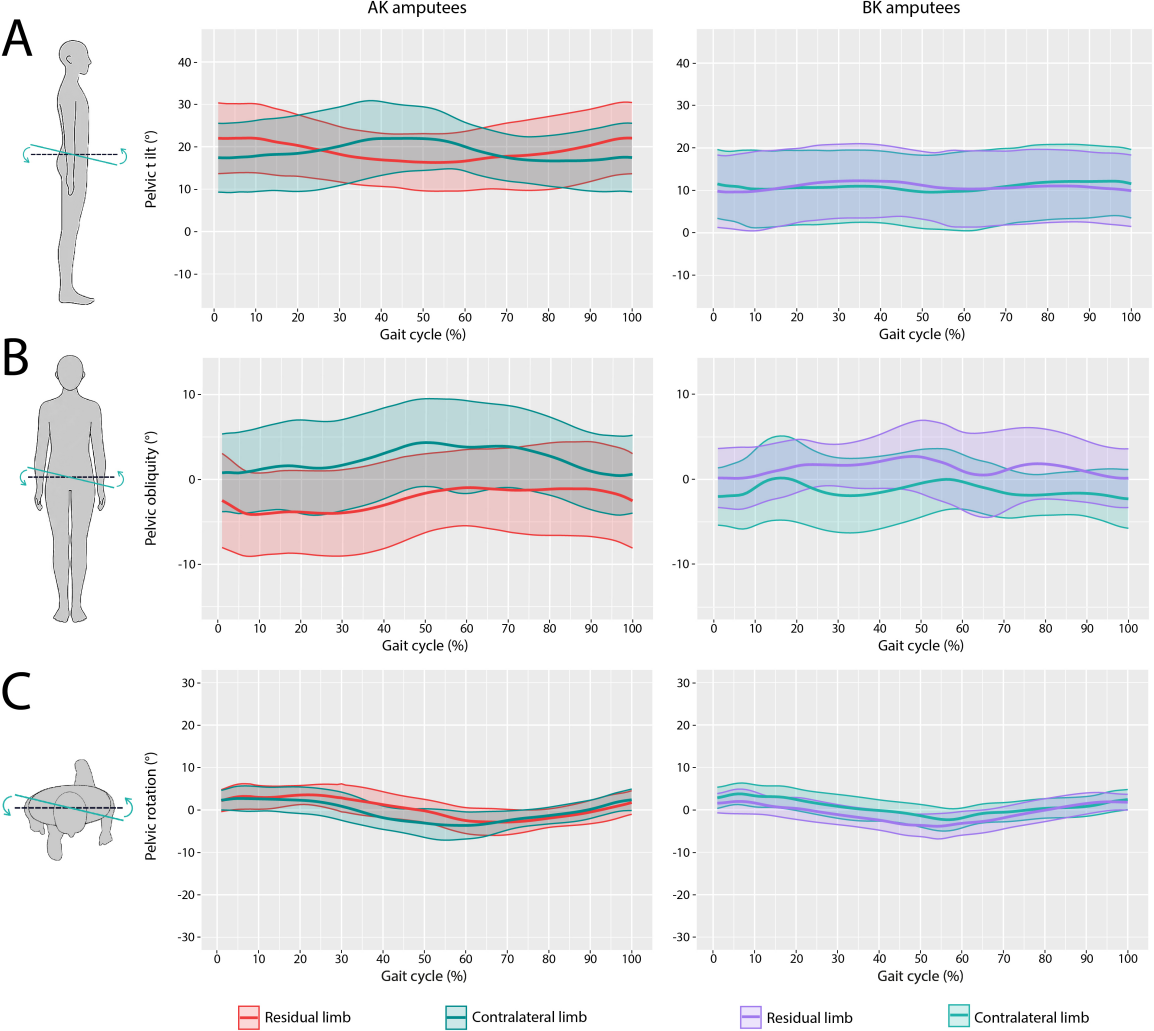
Pelvic kinematics. This figure illustrates pelvic motion in three planes: (A) pelvic tilt, which represents anterior and posterior movement; (B) pelvic obliquity, indicating upward and downward tilting of the pelvis; (C) pelvic rotation, showing rotational movement around the vertical axis. Each curve corresponds to the movement of a limb during gait, and the shaded area represents the standard deviation, indicating variability among subjects. AK: above-knee; BK: below-knee.

The variables that exhibited significant differences across the groups included 2MWT, stride length, cadence, and the symmetry index, with AK amputees showing lower values, except for stride length.

Similar stride length between the amputated and sound limbs were reflected in kinematic behavior of the pelvis, as no alterations in pelvic rotation were observed for either level of amputation. Additionally, a lower SD was recorded throughout the entire cycle, with no considerable variation from the reference values for nonamputated individuals by Lewis et al [20]. The largest SD was observed for pelvic obliquity, which deviates from the trend observed in nonamputated individuals, with a reference range of 0°—5.6° [21]. This deviation was found to be more pronounced for AK amputees and lesser among BK amputees in the sound limb.

Regarding pelvic tilt, substantial alterations were observed in AK amputees, who exhibited a more pronounced anterior tilt throughout the cycle with an inverse relationship between both limbs. For BK amputees, a more conventional gait pattern with little difference between the amputated and sound limb was observed, although a large SD remained high.

## Discussion

### Principal Results

This study revealed significant variations in pelvic kinematics based on the amputation level. Most amputees favored their intact limb for weight-bearing. No major differences in walking speed or propulsion indices were found between AK and BK amputees; however, the AK amputees had lower scores in the 2MWT, cadence, symmetry index, and stride length, along with greater deviations in pelvic tilt and obliquity compared to nonamputated individuals. Accurate reference values for BWD, gait symmetry, and pelvic kinematics are crucial for improving diagnostic precision and customizing prosthetic solutions, given the variability in amputation conditions.

### Comparison With Prior Work

Regarding BWD, BK amputees showed better performance than AK amputees; however, both groups still exhibited asymmetry in weight-bearing between the prosthetic and sound limb. This may be attributed to the fact that most amputees tended to load the intact limb more heavily, than the amputated limb, regardless of the level of amputation. Asymmetrical BWD between sound and amputated limb was also reported by Fontes et al [1]. Factors such as pain, discomfort, and insecurity have been suggested as potential contributors to body weight asymmetry; however, it is worth noting that the patients participating in this study did not explicitly report such conditions. Nevertheless, we cannot rule them out at a subconscious level.

Conversely, discrepancies in the 2MWT, cadence, symmetry index and stride length findings may be attributed to the fact that the presence of the knee joint in BK amputees is related to proprioception and the placement of the center of gravity, leading to reduced energy expenditure during walking. This allows BK amputees to cover longer distances and take more steps per minute, contributing to a more adequate symmetry [22]. Additionally, a longer residual limb provides improved suspension and a greater lever arm for prosthetic control. Preserving the entire femur also enables the amputees to bear all their weight on the distal end of their amputated limb [23].

Regarding velocity, the obtained values closely align with those reported in the literature for amputees, ranging from 0.5 to 0.99 m/s [24,25], which is not far from the range for nonamputated individuals, which lies between 1.2 and 1.4 m/s [25] .

No significant difference in propulsion and quality indices could be explained by the compensatory adjustments made during the gait cycle; for example, the support time using the prosthetic device is shortened when compared to established values [26].

Moreover, the observed asymmetric gait differences confirm that lower limb amputees often exhibit asymmetrical gait patterns, which could lead to long-term health problems and ultimately affect their quality of life [27]. Symmetry measurement could provide clinically useful means of reliably capturing lower extremity motion asymmetries that are not evident while using typical temporal-spatial gait parameters or not easily quantifiable during physician observation [27].

Amputees tend to maximize the capabilities of the sound limb in order to counteract the limitations of the prosthetic device. However, these compensatory mechanisms can have serious consequences that could lead to new physical disabilities [28]. Investigating these strategies in depth presents an interesting avenue for research.

With respect to the shorter stride length observed in BK amputees, it could be linked to the type of suspension used. In this study, all BK amputees used a knee brace, while the majority of AK amputees used an atmospheric suspension system, which directly influences gait stability and, consequently, results in an increased stride length during walking [29].

Changes in pelvic kinematics may be attributed to compensatory adjustments of different prosthetic components. Particularly, pelvic tilt might be influenced by stability in re-establishing the center of gravity, and pelvic obliquity could be influenced by prosthetic adjustments such as suspension system or prosthesis length [30].

### Limitations

This study has several limitations. The sample size was relatively small, which may impact the generalizability of the findings. The study focused on a specific demographic, potentially limiting broader applicability. Measurement techniques had constraints that may introduce potential errors. Regarding velocity, participants were instructed to walk at a self-determined pace in a controlled environment, which could have influenced the outcomes.

### Conclusions

The differences in gait variables among amputees highlight the need for tailored approaches based on the amputation level. Symmetry and quality indices facilitate comparisons and can be applied to other populations with gait disorders. The values reported in this study provide reference levels that support diagnosis and prosthetic fitting. The increasing use of inertial sensors and pressure platforms reinforces their role as accessible clinical tools outside specialized gait laboratories.

## Abbreviations

2MWT: two-minute walking test
AK: above-knee
AP: anteroposterior
BK: below-knee
BWD: body weight distribution
